# Association between homocysteine levels and hypertension prevalence as well as all-cause mortality and cardiovascular mortality among hypertensive patients: A population-based study

**DOI:** 10.1371/journal.pone.0330267

**Published:** 2025-08-12

**Authors:** Wenna Wang, Hao Lu, Peng Pu, Huan Yin, Linlin Huang

**Affiliations:** 1 Department of Cardiology, Chognqing Red Cross Hospital (People’s Hospital of Jiangbei District), Chongqing, China; 2 Department of General Practice, The First Affiliated Hospital of Chongqing Medical University, Chongqing, China; 3 Department of Cardiology, The First Affiliated Hospital of Chongqing Medical University, Chongqing, China; Universidad de Murcia, SPAIN

## Abstract

**Background:**

Currently, some studies have investigated the relationship between homocysteine (Hcy) levels and hypertension. However, within the population of individuals with hypertension, there is still a lack of relevant research data. Therefore, we utilized data from the National Health and Nutrition Examination Survey (NHANES) to explore the associations between Hcy levels and hypertension prevalence, all-cause mortality, and cardiovascular mortality, in order to understand the role of Hcy levels in the control, assessment, and treatment of hypertension.

**Methods:**

In this study, the data utilized were derived from NHANES, which collected data from 1999 to 2006. A total of 7680 eligible participants were ultimately included. To explore the associations between Hcy levels and hypertension prevalence as well as blood pressure, a weighted multivariate linear regression model and restricted cubic splines were employed to adjust for confounding factors. Additionally, we conducted subgroup analyses to observe the associations between Hcy levels and hypertension prevalence, systolic blood pressure(SBP), and diastolic blood pressure (DBP) in different subgroups. Lastly, we employed Kaplan-Meier estimates and Cox proportional hazards regression models for survival analysis, elucidating the relationship between hyperhomocysteinemia (HHcy) and the risks of all-cause mortality and cardiovascular mortality among hypertensive patients.

**Results:**

The average age of all participants was 44.82 years, with 51.8% being female. After adjusting for relevant covariates, a positive correlation between Hcy and the risk of hypertension was identified (OR=1.04, 95% *CI*: 1.02–1.07, *P* < 0.001). Multivariate linear regression results indicated a positive correlation between HHcy and blood pressure levels (SBP: β = 0.2, 95% *CI*: 0.10–0.30, *P* < 0.001; DBP: β = 0.09, 95% *CI*: 0.01–0.17, *P* < 0.05). Furthermore, restricted cubic spline(RCS) curve analysis revealed a nonlinear positive correlation between SBP and DBP with Hcy levels. Survival analysis results demonstrated that when blood Hcy concentrations exceeded 10 μmol/L, patients with hypertension experienced significantly increased all-cause and cardiovascular mortality rates (P < 0.001).

**Conclusion:**

Our research validates Hcy as an independent risk factor for hypertension, further confirming a nonlinear positive correlation between blood pressure and Hcy levels. HHcy was associated with small increases SBP and DBP proportional to the degree of homocysteine elevation. Additionally, HHcy was a high-risk factor for all-cause and cardiovascular mortality in hypertensive patients. This may provide new insights into the management and treatment of hypertension.

## 1. Introduction

Hypertension, the most prevalent cardiovascular disease (CVD) globally, has emerged as a major public health concern, consistently drawing significant attention from researchers. Hypertension accounts for one-third of global deaths. Epidemiological studies indicate that 59% of women and 49% of men suffered from hypertension in 2019 [1]. Previous studies have shown that vascular wall thickening and increased peripheral vascular resistance caused by endothelial cell injury are crucial factors in the development and progression of hypertension [2]. Therefore, factors that may lead to these pathological changes can be considered as risk factors for hypertension. Currently, Hcy is considered a potential risk factor for hypertension and is notably prominent among various risk factors.

Hcy is a non-essential sulfur-containing amino acid with cytotoxicity, and unhealthy lifestyles such as chronic heavy alcohol consumption, caffeine intake, long-term smoking, drinking strong tea, and lack of exercise may lead to elevated Hcy levels [3].HHcy is considered a risk factor for CVD and stroke [4,5].Hypertension is closely linked to stroke. The CSPPT study and relevant analyses have confirmed that adding low-dose folic acid to antihypertensive drugs can significantly reduce Hcy levels and stroke incidence in hypertensive patients [6,7]. After defining HHcy levels as ≥14.5 μmol/L in a cohort study on Hcy and CVD – related deaths in CVD populations, Liu et al. [8] found a positive correlation between HHcy and CVD deaths through subgroup analyses. However, there is currently a lack of research data on Hcy in hypertension-specific populations, including discussions on the safe upper limit of Hcy, which is of great significance for the optimal treatment of HHcy, especially in combination with antihypertensive therapy.

In this study, we utilized the large sample data from NHANES between 1999 and 2006, in controlling for multiple confounding factors, to simultaneously investigate the associations between Hcy levels and hypertension prevalence, all-cause mortality, and cardiovascular mortality. The aim is to expand the evidence base for the relationship between Hcy and hypertension and its mortality risks, providing new evidence for future diagnosis and treatment of hypertension.

## 2. Materials and methods

### 2.1 Data source and population

**Fig 1:**
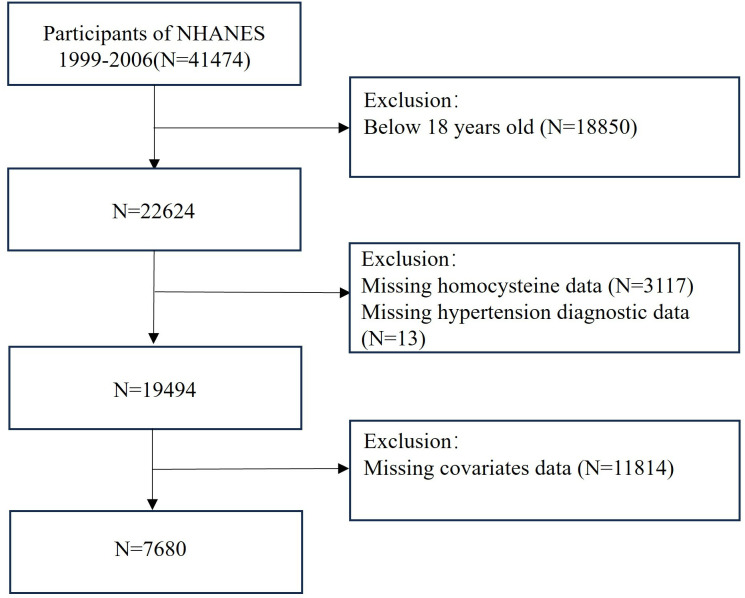
Flowchart for selecting analyzed participants.

All data utilized in this research were derived from the NHANES database. Approval for this study was granted by the National Center for Health Statistics Research Ethics Review Board in the United States. NHANES annually surveys approximately 5000 individuals spanning various age groups. Participants must sign a consent form agreeing to participate in home interviews, physical examinations, and laboratory tests. Publicly available data have been anonymized and do not contain personal information. This study utilized data from 41474 participants spanning the years 1999–2006, and linked it to mortality data from the National Death Index (NDI) collected from January 1999 to December 2019. After excluding participants under 18 years old, those lacking homocysteine and hypertension diagnosis data, as well as those missing at least one covariate, 7680 participants were ultimately included. The flowchart of the screening process is shown in Fig 1.

### 2.2 Assessment of hypertension

Blood pressure examiners, certified through the training program of Shared Care Research and Education Consulting, measured blood pressure using standardized procedures with mercury sphygmomanometers in mobile examination centers. Measurements were taken in a seated position, primarily using the right arm unless there were special circumstances. After participants rested for 5 minutes, their blood pressure was measured three consecutive times, and the average of these three readings was used for subsequent analysis. Hypertension was defined as SBP ≥ 140 mmHg and/or DBP ≥ 90 mmHg, or self-reported physician-diagnosed hypertension or the use of antihypertensive medications [9].

### 2.3 Measurement of plasma homocysteine

Plasma Hcy was measured using the “Abbott Homocysteine assay”, where Hcy is first converted to S-adenosyl-homocysteine by the action of dithioerythritol, and then, in the presence of added adenosine, S-adenosyl-homocysteine (SAH) hydrolase is applied to catalyze the conversion of homocysteine to SAH. Subsequently, a fluorescently labeled S-adenosyl-homocysteine analog and specific monoclonal antibodies are added, and finally, the Abbott AxSym instrument is used to calculate the plasma tHcy concentration using a pre-stored calibration curve [10,11]. Based on previous studies, we defined plasma Hcy concentrations exceeding 10 μmol/L as elevated [12].

### 2.4 Covariates assessment

Our study includes covariates that may influence hypertension, including age (years old), gender (male/female), race (Mexican American, non-Hispanic white, non-Hispanic black, other),marital status (married, single, living with a partner), education level (below high school, high school, above high school), family income-to-poverty ratio (PIR), physical activity level (METs), diabetes history (yes, no, borderline), serum cotinine level (ng/ml), and body mass index (kg/m^2^). Hyperlipidemia is defined as serum total triglycerides (TG) ≥150 mg/dL (1.7 mmol/L) or serum total cholesterol (TC) ≥200 mg/dL (5.18 mmol/L), with low-density lipoprotein cholesterol (LDL) ≥130 mg/dL (3.37 mmol/L) or high-density lipoprotein cholesterol (HDL) <40mg/dL (1.04 mmol/L) for males, 50 mg/dL (1.30 mmol/L) for females, or the use of lipid-lowering medications [13]. Cardiovascular disease is defined as having any of the following conditions reported by a physician: congestive heart failure, coronary heart disease, angina, heart attack, or stroke. Alcohol (g), sodium (mg), and caffeine intake (mg) were averaged over two days if dietary intake data were complete for both days, otherwise, the intake from the first day was used. In this study, PIR was categorized into <1, 1–3, and >3. More detailed explanations of all variables can be obtained from the NHANES database website.

### 2.5 Statistical analysis

Following Centers for Disease Control and Prevention (CDC) guidelines, we applied fasting subsample weights (WTSAF4YR for 1999–2002 and WTSAF2YR for 2003–2006) to account for the stratified, multistage probability sampling of NHANES, ensuring nationally representative estimates. Continuous variables are presented as means (standard error, SE), while categorical variables are presented as proportions (SE). Baseline comparisons were conducted between groups stratified by the presence or absence of hypertension. Chi-square tests were used to determine p-values for categorical variables, and t-tests were employed for continuous variables. On the other hand, Hcy was converted into quartiles for baseline comparison analysis, with p-values for categorical variables determined using Chi-square tests and p-values for continuous variables determined using Kruskal-Wallis rank-sum tests. In various covariate-adjusted scenarios, this study used three logistic regression models to assess the association between Hcy and hypertension prevalence. Weighted multivariable linear regression models were applied to evaluate the relationships of SBP and DBP with Hcy. After covariate adjustment, RCS was used to evaluate the nonlinear relationships. Finally, subgroup analysis was conducted in this study to address potential confounding factors, calculating the significance of effects and interactions of hypertension prevalence, SBP, and DBP across different subgroups, and represented by a forest plot. The data were analyzed using R software (R 4.4.2). Statistical significance was defined as a P value of < 0.05.

## 3. Results

### 3.1 Baseline characteristics of participants

The study included demographic, examination, laboratory, and questionnaire data from selected individuals in the NHANES survey conducted between 1999 and 2006. The weighted distribution of baseline characteristics, grouped by the presence or absence of hypertension, is presented in Table 1. In our study, the primary population among the selected individuals was non-Hispanic Whites, with a mean age of 44.82 years. Statistically significant differences were observed in age, BMI, serum cotinine levels, mean systolic and DBP, race, marital status, PIR, histories of hyperlipidemia, cardiovascular disease, diabetes, sodium intake, and serum Hcy distribution (P < 0.05). However, differences in the distribution of gender, physical activity level, caffeine intake, and alcohol consumption were not statistically significant (**P* *> 0.05). Additionally, we categorized serum Hcy into quartiles (Q1 to Q4), and the baseline analysis tables are provided in S1 Table.

**Table 1:** Clinical characteristics of the study population classified by hypertension.

Variable	Total	No	Yes	*P* value
	(*n* = 7680)	(*n* = 4945)	(*n* = 2735)	
Age(years)	44.82(0.37)	40.44(0.32)	54.20(0.56)	< 0.001
BMI (kg/m2)	27.96(0.13)	27.07(0.14)	29.84(0.17)	< 0.001
Cotinine (ng/ml)	61.74(2.68)	64.50(3.02)	55.81(2.98)	0.004
SBP (mmHg)	121.29(0.34)	114.83(0.25)	135.12(0.50)	< 0.001
DBP (mmHg)	72.08(0.24)	69.97(0.19)	76.59(0.43)	< 0.001
Sex (%)				0.16
Female	49.02(0.02)	49.62(0.64)	47.73(1.08)	
Male	50.98(0.02)	50.38(0.64)	52.27(1.08)	
Race (%)				< 0.001
Mexican American	6.02(0.01)	6.97(0.64)	3.97(0.57)	
Non-Hispanic Black	8.07(0.01)	7.45(0.73)	9.37(1.01)	
Non-Hispanic White	77.91(0.05)	77.08(1.50)	79.70(1.37)	
Other Race	8.01(0.01)	8.50(0.94)	6.96(0.72)	
Marriage (%)				< 0.001
Living with partner	6.93(0.01)	8.03(0.58)	4.58(0.42)	
Married	61.50(0.03)	59.90(1.12)	64.91(1.41)	
Single	31.57(0.01)	32.07(1.03)	30.51(1.25)	
Education (%)				< 0.001
High school	24.83(0.02)	23.83(1.05)	26.97(1.12)	
Less than high school	13.85(0.01)	12.42(0.71)	16.91(0.99)	
More than high school	61.32(0.03)	63.75(1.30)	56.12(1.43)	
Pir (%)				0.004
< 1	9.83(0.01)	10.69(0.71)	7.98(0.66)	
> 3	57.19(0.03)	56.87(1.60)	57.88(1.53)	
1-3	32.98(0.02)	32.44(1.24)	34.14(1.35)	
Hyperlipidemia (%)				< 0.001
No	28.80(0.01)	34.45(0.94)	16.71(1.11)	
Yes	71.20(0.04)	65.55(0.94)	83.29(1.11)	
CVD (%)				< 0.001
No	94.07(0.04)	97.00(0.29)	87.79(0.64)	
Yes	5.93(0.00)	3.00(0.29)	12.21(0.64)	
Diabetes (%)				< 0.001
Borderline	1.08(0.00)	0.56(0.11)	2.19(0.40)	
No	93.62(0.04)	96.44(0.36)	87.60(0.78)	
Yes	5.30(0.00)	3.00(0.32)	10.21(0.63)	
Physical activity (METs)	970.79(35.98)	976.78(41.74)	957.95(43.74)	0.7
Caffeine intake(mg)	198.17(4.79)	197.10(5.28)	200.46(5.63)	0.53
Alcohol intake(g)	12.18(0.61)	12.52(0.78)	11.43(0.88)	0.35
Na intake(mg)	3568.89(26.14)	3626.92(31.91)	3444.60(39.82)	< 0.001
Hcy(umol/l)	8.45(0.06)	8.01(0.07)	9.37(0.09)	< 0.001

### 3.2 Relationship between Hcy levels and SBP

We used weighted linear regression models to examine the correlation between serum Hcy levels and mean SBP, and separately fitted models for continuous Hcy and Hcy in quartiles (Table 2), to delve into the impact of Hcy on hypertension prevalence across different level ranges. In Model 1, for every 1 umol/L increase in Hcy, SBP increased by 0.75 (95% *CI*, 0.56–0.93) mmHg. Model 2 was adjusted for age, gender, race, and education level. Model 3 further adjusted for sodium intake, serum cotinine concentration, body mass index, family poverty index, history of diabetes, hyperlipidemia, and cardiovascular disease, in addition to the adjustments made in Model 2. The β values for Models 2 and 3 were 0.18 (95% *CI*, 0.08–0.28) and 0.2 (95% *CI*, 0.10–0.30), respectively. Furthermore, after adjusting for all confounding factors, the Hcy Q4 group showed a statistically significant difference compared to the Q1 group, with a *β* value of 2.18 (95% *CI*, 0.81–3.56).

**Table 2:** Multivariate weighted linear model analysis reveals the association between Hcy and SBP.

	Model1	Model2	Model3
	β(95%*CI*) *P* value	β(95%*CI*) *P* value	β(95%*CI*) *P* value
Hcy	0.75(0.56,0.93) <0.001	0.18(0.08, 0.28) <0.001	0.2(0.10, 0.30) <0.001
Hcy			
Q1	ref	ref	ref
Q2	4.18(2.94, 5.43) <0.001	1.1(−0.01, 2.21)0.05	0.99(−0.10, 2.07)0.07
Q3	6.79(5.51, 8.07) <0.001	1.25(−0.05, 2.54)0.06	1.11(−0.12, 2.35)0.08
Q4	11.3(10.05,12.54) <0.001	2.26(0.82, 3.70)0.003	2.18(0.81, 3.56)0.003
*P* for trend	<0.001	<0.001	<0.001

Model 1 is unadjusted. Model 2 was adjusted for age, gender, race, and education level. Model 3 adjusted for age, gender, race, and education level, sodium intake, serum cotinine concentration, body mass index, family poverty index, history of diabetes, hyperlipidemia, and cardiovascular disease.

### 3.3 The relationships between Hcy levels and DBP

The weighted linear regression model was used to further discuss the correlation between serum Hcy levels and mean DBP (Table 3). The results revealed a statistically significant correlation between Hcy levels and DBP after adjusting for all confounding factors. In Model 3, an increase of 1 umol/L in Hcy was associated with a 0.09 mmHg (95% *CI*, 0.01–0.17) increase in DBP. Model 3 was adjusted for age, gender, race, education level, sodium intake, serum cotinine concentration, body mass index, household poverty index, history of diabetes, hyperlipidemia, and cardiovascular disease. When Hcy was categorized into quartiles (Q1-Q4), Model 3 showed statistically significant β values of 1.19 (0.26, 2.11), 1.49 (0.58, 2.39), and 1.04 (0.03, 2.05) for Q2, Q3, and Q4, respectively, compared to Q1. The trend tests for all three models were statistically significant.

**Table 3:** Multivariate weighted linear model analysis reveals the association between Hcy and DBP.

	Model1	Model2	Model3
	β(95%*CI*) *P* value	β(95%*CI*) *P* value	β(95%*CI*) *P* value
Hcy	0.17(0.09,0.25) <0.001	0.04(−0.03, 0.12)0.27	0.09(0.01, 0.17)0.03
Hcy			
Q1	ref	ref	ref
Q2	2.32(1.39,3.25) <0.001	1.34(0.45, 2.23)0.004	1.19(0.26, 2.11)0.01
Q3	3.21(2.40,4.02) <0.001	1.63(0.74, 2.51) <0.001	1.49(0.58, 2.39)0.002
Q4	2.94(2.11,3.76) <0.001	0.87(−0.16, 1.89)0.09	1.04(0.03, 2.05)0.04
*P* for trend	<0.001	<0.001	<0.001

Model 1 is unadjusted. Model 2 was adjusted for age, gender, race, and education level.Model 3 adjusted for age, gender, race, and education level, sodium intake, serum cotinine concentration, body mass index, family poverty index, history of diabetes, hyperlipidemia, and cardiovascular disease.

### 3.4 Restricted cubic spline curve analysis

**Fig 2:**
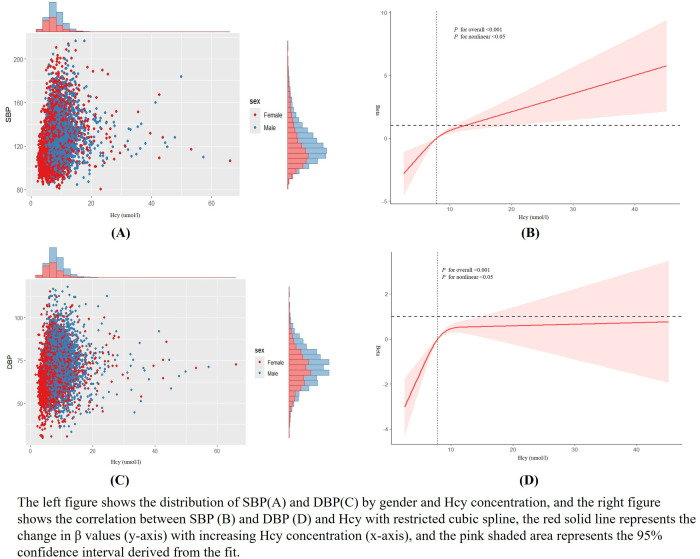
The RCS shows the relationship between Hcy and SBP and DBP.

We visualized the distribution relationship between SBP (Fig 2A) and DBP (Fig 2C) and Hcy by gender using marginal distribution histograms. It can be seen that female hypertensive patients are more distributed in the low-level Hcy range. After adjusting for age, gender, race, education level, sodium intake, serum cotinine concentration, body mass index, household poverty index, history of diabetes, hyperlipidemia, and cardiovascular disease, the restricted cubic spline analysis revealed a nonlinear relationship between Hcy and both SBP (Fig 2B) and DBP (Fig 2D), with nonlinear *P* values < 0.05. With the increase of Hcy concentration, SBP and DBP showed a positive correlation with Hcy levels (*P* < 0.001).

### 3.5 Relationship between Hcy levels and hypertension prevalence

We employed a weighted logistic regression model to investigate the correlation between Hcy and hypertension prevalence (Table 4). Based on the results, we observed a statistically significant positive correlation between Hcy levels and hypertension prevalence in all three models. In Model 3, after adjusting for age, gender, race, education level, sodium intake, serum cotinine concentration, body mass index, household poverty index, history of diabetes, hyperlipidemia, and cardiovascular disease, each unit increase in Hcy was associated with a 4% increase in the risk of hypertension (95% *CI*, 1.02–1.07) mmHg. When Hcy was categorized into quartiles (Q1-Q4), the hypertension prevalence risk significantly increased in the Hcy Q4 group compared to the Q1 group, with an OR of 1.50 (95% *CI*, 1.22–1.85).

**Table 4:** Multivariate weighted linear model analysis reveals the association between Hcy and hypertension prevalence.

	Model1	Model2	Model3
	OR (95%*CI*) *P* value	OR (95%*CI*) *P* value	OR (95%*CI*) *P* value
Hcy	1.25(1.14,1.36) <0.001	1.04(1.02,1.06) <0.001	1.04(1.02,1.07) <0.001
Hcy			
Q1	ref	ref	ref
Q2	1.57(1.31,1.89) <0.001	1.19(0.97,1.45)0.10	1.18(0.95,1.47)0.12
Q3	2.09(1.78,2.45) <0.001	1.27(1.07,1.50)0.01	1.23(1.03,1.46)0.02
Q4	3.52(2.95,4.19) <0.001	1.55(1.24,1.93) <0.001	1.50(1.22,1.85) <0.001
*P* for trend	<0.001	<0.001	<0.001

Model 1 is unadjusted. Model 2 was adjusted for age, gender, race, and education level. Model 3 adjusted for age, gender, race, and education level, sodium intake, serum cotinine concentration, body mass index, family poverty index, history of diabetes, hyperlipidemia, and cardiovascular disease.

### 3.6 Subgroup analysis

**Fig 3:**
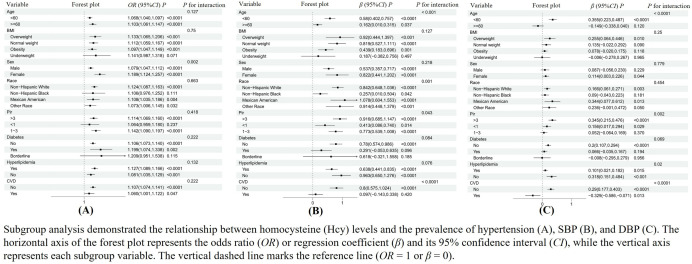
Subgroup analysis of the relationship between Hcy levels and the prevalence of hypertension, SBP, and DBP.

To further validate the stability of the results from the multiple linear regression analysis, we further analyzed the stratified relationship between Hcy levels and hypertension prevalence, SBP, and DBP in specific subgroups, including age, gender, race, PIR, BMI, diabetes history, and hyperlipidemia history (Fig 3). During the stratified analysis of hypertension prevalence, we found no interaction between Hcy levels and age, PIR strata, BMI strata, diabetes history, hyperlipidemia history, or cardiovascular disease history. However, compared to males, females exhibited a higher risk of 1.189 (95% *CI*: 1.124–1.257), with statistical significance (Fig 3A). Based on the results, we found that SBP increased with increasing Hcy concentrations across age strata, gender strata, PIR strata, and hyperlipidemia history strata, with statistical significance (*P* < 0.05) (Fig 3B).Additionally, we found that age, race, and cardiovascular disease history may have interactive effects with SBP (interaction p < 0.05). After stratified analysis of DBP, we found that age, PIR, hyperlipidemia history, and cardiovascular disease history may have interactive effects with DBP (interaction p < 0.05) (Fig 3C).

### 3.7 Survival analysis

**Fig 4:**
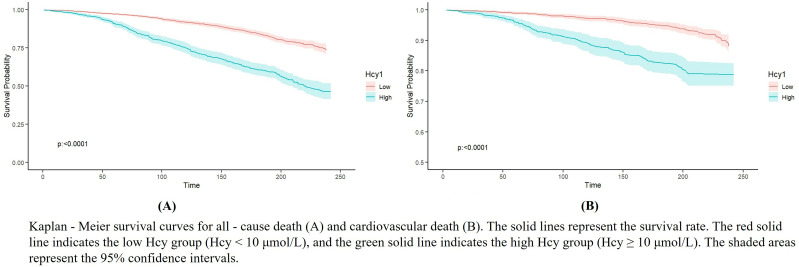
Kaplan-Meier curves of all-cause mortality and cardiovascular mortality in hypertensive patients.

The Kaplan-Meier curve (Fig 4) showed that HHcy was associated with all-cause mortality (Fig 4A) and cardiovascular mortality (Fig 4B) in hypertensive patients (*P* < 0.05). After adjusting for factors such as age, gender, race, education level, sodium intake, serum cotinine concentration, BMI, PIR, diabetes history, hyperlipidemia history, and cardiovascular disease history in Model III, the multivariate Cox regression analysis showed that HHcy was consistently associated with increased risks of all-cause mortality and cardiovascular mortality in hypertensive patients (HR = 1.469, 95% *CI*: 1.266–1.704, **P* *< 0.001); (HR = 1.399, 95% *CI*: 1.029–1.902, **P* *< 0.05) (Table 5).

**Table 5:** Multivariate weighted COX proportional regression model analysis reveals the association between Hcy and all-cause and cardiovascular mortality in hypertensive patients.

		Risk of all-cause mortality	Risk of cardiovascular mortality
	Hcy	HR (95%*CI*) *P* value	HR (95%*CI*) *P* value
Model I	Low	ref	ref
	High	2.828(2.422,3.301) <0.001	3.134(2.312,4.247) <0.001
Model II	Low	ref	ref
	High	1.643(1.403,1.924) <0.001	1.577(1.161,2.142)0.004
Model III	Low	ref	ref
	High	1.469(1.266,1.704) <0.001	1.399(1.029,1.902)0.032

Model I is unadjusted. Model II was adjusted for age, gender, race, and education level. Model III adjusted for age, gender, race, and education level, sodium intake, serum cotinine concentration, body mass index, family poverty index, history of diabetes, hyperlipidemia, and cardiovascular disease.

## 4. Discussion

In this study, we employed a highly representative individual sample from the NHANES conducted between 1999 and 2006 to investigate the relationship between Hcy levels and the risk of hypertension among adults in the United States. After adjusting for relevant covariates, we found a positive correlation between Hcy and the risk of hypertension. The AUC in the ROC analysis was calculated to be 0.664 (95% CI: 0.651−0.677). Through RCS analysis, we found a nonlinear positive correlation between systolic and DBP and Hcy levels. Further, stratified analysis showed that this positive correlation was more pronounced in individuals under 60 years old and those without a history of cardiovascular disease. Lastly, survival analysis results showed that high Hcy was associated with decreased survival rates among hypertensive patients. It is a high-risk factor for all-cause and cardiovascular mortality among hypertensive patients.

Hcy is an intermediate product of the methionine cycle and cysteine metabolism. It is a non-essential amino acid with cytotoxicity that cannot be directly synthesized by the human body [14].After absorption, methionine is converted into s-adenosylmethionine in the body. Under the catalysis of methyltransferase, it loses a methyl group. Subsequently, it is catalyzed by s-adenosylhomocysteine hydrolase to remove adenosine, forming Hcy [15].Hcy metabolism is closely related to folic acid. Folic acid, an oxidized synthetic analog, is often used as a pro-vitamin. It can be effectively converted into active L-5-methyltetrahydrofolate in the liver when the daily dose doesn’t exceed 400−800 µg. L-5-methyltetrahydrofolate, a folate metabolite in the body, serves as the methyl donor for Hcy remethylation, promoting Hcy metabolism [16,17].Since 1997 folic acid has been used in the US for food fortification, and homocysteine levels have dropped significantly [18]. Folic acid intervention in the US would likely be less effective that in non-fortified countries such as Europe and China because the bulk of the US population (and this NHANES cohort) has adequate folate [19]. In the US riboflavin (B2), B6, and B12 can be combined with L-5-methyltetrahydofolate (e.g., Metafolin) for effective Hcy reduction, with improvement in BP [20].In Xie’s study, the correlation between serum folate levels and the prevalence of elderly diastolic hypertension was explored. Higher serum folate levels were found to be associated with a lower prevalence of diastolic hypertension in the elderly, which is closely related to folate metabolism’s ability to reduce blood Hcy levels [21]. Our study further confirms this conclusion. As Hcy levels increase, the risk of elevated DBP also shows a gradual increase (*P* < 0.001), but due to the wide confidence intervals, this finding requires further validation with larger sample data.

In hypertension’s pathogenesis, vascular endothelial function is essential. As a protective vascular barrier, it regulates vascular tone, coagulation, inflammation and permeability [22,23]. Conversely, endothelial dysfunction can prompt inflammatory responses and trigger a cascade of reactions like vascular remodeling [24]. Studies have shown that Hcy can damage endothelial cells. Causing subendothelial edema, leading to the accumulation of fibrous tissue and an increase in interstitial collagen fibers. On the other hand, Hcy can increase reactive oxygen species (ROS) and oxidative stress, alter DNA and gene expression, increase the procoagulant activity of coagulation factors, and inhibit the expression of anticoagulant molecules. Ultimately, this leads to thickening of the vascular wall and vascular dysfunction [25–27]. This may be the mechanism underlying the association between Hcy and hypertension.

Previous studies by Liu et al. [8] established a significant association between elevated HHcy levels and increased risks of all-cause and cardiovascular mortality among cardiovascular patients, including those with coronary heart disease, cerebrovascular disease, and heart failure. They identified inflection points at 14.5 μmol/L for all-cause mortality and 14.6 μmol/L for cardiovascular mortality. However, Smith and Refsum [28] concluded that Hcy levels ≤10 μmol/L may represent a safe threshold. Our analysis of hypertension-specific cohorts demonstrated that Hcy levels exceeding 10 μmol/L significantly increase mortality risks in hypertensive patients. This finding validates the 10 μmol/L threshold and underscores the importance of early intervention in this population. Compared to Liu’s study, our research extended to individuals aged 18 and older, encompassing a broader and more diverse cohort. Our findings further confirm that HHcy serves as a risk factor for disease development and reduced survival rates in hypertensive populations. Survival analysis results indicated that 10 μmol/L represents a safer threshold for hypertensive individuals. Considering the established relationships among hypertension, coronary artery disease, cerebrovascular disease, and stroke, it is plausible that safe upper limits for Hcy may vary across different disease states.

In the baseline analysis of our study, where Hcy levels were grouped by quartiles, it was found that the proportion of males was higher in the higher quartiles of Hcy levels compared to the lower quartiles (P < 0.001). This may be due to higher creatine synthesis levels in males and the estrogen-mediated reduction of Hcy levels in females [29]. As the Hcy level increases, the average age of the population gradually rises (P < 0.0001), which is consistent with previous observations [30]. This may be due to the reduced absorption of vitamins B2, B6, and B12 with increasing age. The results of this study further confirm that the Hcy level is an independent risk factor for hypertension, as previously found in other studies [31].

The strengths of this study include its large sample size and long-term follow-up. It evaluates the relationship between Hcy and hypertension prevalence and mortality in a large sample size. Additionally, the NHANES dataset, which is derived from a folate-fortified population, has been extensively studied, validated, and widely recognized. However, in our study, we need to consider several potential limitations. Firstly, this study cannot infer the causal relationship between Hcy and hypertension. Larger sample-sized prospective studies are needed to further explore the causal relationship between these two variables. Furthermore, the data samples in this study are sourced from 1999–2006, as only these years have publicly available Hcy data, which imposes certain limitations on our sample size. The folic acid fortification measures implemented in the United States in 1997 effectively reduced the level of homocysteine. Therefore, this study may not be directly applicable in countries that have not implemented folic acid fortification, such as Western Europe or China. Finally, due to the retrospective nature of the study, there may still be confounding factors that have not been considered, although we have tried to control them as much as possible. Additionally, due to the missing data of covariates, we excluded nearly half of the samples, which may weaken the representativeness of this study to some extent. Despite these limitations, our study results can expand our understanding of the relationship between Hcy and hypertension, as well as its mortality risk, increase evidence-based support, and provide new insights and clues for future research on predicting adverse events in hypertensive patients.

## 5. Conclusion

This study confirms that Hcy is an independent risk factor for hypertension, and there is a nonlinear positive correlation between blood pressure and Hcy levels. Additionally, the study confirmed that Hcy is associated with all-cause and cardiovascular mortality in individuals diagnosed with hypertension. In hypertension management, assessing Hcy levels can facilitate the identification of high-risk hypertensive patients. Furthermore, this threshold can serve as an intervention target to reduce mortality risk.

## Supporting information

S1 Table Clinical characteristics of the study population by Hcy quartiles. (DOCX)

S1 File Original R code. (DOCX)
