# Supplementary material for: Association between homocysteine levels and hypertension prevalence as well as all-cause mortality and cardiovascular mortality among hypertensive patients: A population-based study
Source: PLoS One. 2025 Aug 12;20(8):e0330267. doi: 10.1371/journal.pone.0330267 (PMC12342259; doi:10.1371/journal.pone.0330267)
Supplement: S1 Table — (DOCX) [file pone.0330267.s001.docx]

Supporting Information

# Supplementary Tables

| Variable | Q1(n=1928) | Q2(n=1915) | Q3(n=1921) | Q4(n=1916) | *P* value |
| --- | --- | --- | --- | --- | --- |
| Age(years) | 37.69(0.42) | 42.56(0.43) | 46.48(0.44) | 52.86(0.51) | < 0.0001 |
| BMI (kg/m2) | 27.38(0.24) | 27.84(0.18) | 28.19(0.17) | 28.41(0.18) | 0.001 |
| SBP(mmHg) | 115.76(0.43) | 119.95(0.49) | 122.55(0.56) | 127.06(0.55) | < 0.0001 |
| DBP(mmHg) | 69.92(0.36) | 72.24(0.41) | 73.13(0.34) | 72.86(0.35) | < 0.0001 |
| Sex (%) |  |  |  |  | < 0.0001 |
| Female | 76.61(1.23) | 51.25(1.44) | 37.10(1.09) | 32.13(1.23) |  |
| Male | 23.39(1.23) | 48.75(1.44) | 62.90(1.09) | 67.87(1.23) |  |
| Race (%) |  |  |  |  | < 0.0001 |
| Mexican American | 9.09(0.90) | 6.97(0.80) | 4.45(0.56) | 3.60(0.49) |  |
| Non-Hispanic Black | 8.27(0.97) | 7.89(0.74) | 7.88(0.96) | 8.28(0.95) |  |
| Non-Hispanic White | 72.02(1.84) | 77.60(1.61) | 80.99(1.49) | 80.69(1.55) |  |
| Other Race | 10.63(1.33) | 7.54(1.01) | 6.68(0.92) | 7.43(1.00) |  |
| Marriage (%) |  |  |  |  | 0.02 |
| Living with partner | 7.94(1.00) | 7.01(0.61) | 7.47(0.74) | 5.17(0.63) |  |
| Married | 59.60(1.36) | 60.13(1.52) | 63.61(1.36) | 62.54(1.47) |  |
| single | 32.46(1.27) | 32.86(1.51) | 28.92(1.24) | 32.29(1.22) |  |
| Education (%) |  |  |  |  | < 0.001 |
| High school | 22.25(1.79) | 22.88(1.27) | 25.93(1.26) | 28.48(1.55) |  |
| Less than high school | 13.59(0.87) | 12.40(1.01) | 13.85(1.06) | 15.81(1.02) |  |
| More than high school | 64.17(2.13) | 64.72(1.63) | 60.22(1.56) | 55.71(1.63) |  |
| Pir (%) |  |  |  |  | < 0.001 |
| <1 | 12.77(0.97) | 8.74(0.71) | 8.82(0.85) | 9.26(0.91) |  |
| >3 | 53.32(1.82) | 58.27(1.93) | 60.95(1.86) | 55.45(2.03) |  |
| 1-3 | 33.91(1.60) | 32.99(1.95) | 30.23(1.51) | 35.29(1.82) |  |
| Hyperlipidemia (%) |  |  |  |  | < 0.0001 |
| No | 34.91(1.58) | 31.45(1.30) | 25.79(1.32) | 22.98(1.09) |  |
| Yes | 65.09(1.58) | 68.55(1.30) | 74.21(1.32) | 77.02(1.09) |  |
| CVD (%) |  |  |  |  | < 0.0001 |
| No | 98.17(0.35) | 96.41(0.42) | 93.24(0.69) | 88.07(0.81) |  |
| Yes | 1.83(0.35) | 3.59(0.42) | 6.76(0.69) | 11.93(0.81) |  |
| Diabetes (%) |  |  |  |  | < 0.0001 |
| Borderline | 1.10(0.26) | 0.93(0.20) | 0.98(0.28) | 1.36(0.36) |  |
| No | 94.78(0.57) | 95.80(0.50) | 93.28(0.70) | 90.28(0.77) |  |
| Yes | 4.12(0.51) | 3.27(0.44) | 5.75(0.59) | 8.36(0.64) |  |
| Hypertension |  |  |  |  | < 0.0001 |
| No | 80.26(1.20) | 72.10(1.15) | 66.06(0.96) | 53.61(1.43) |  |
| Yes | 19.74(1.20) | 27.90(1.15) | 33.94(0.96) | 46.39(1.43) |  |
| Physical activity (METs) | 915.02(49.69) | 921.57(48.85) | 987.07(56.28) | 1066.84(53.23) | 0.08 |
| Caffeine intake(mg) | 150.80(5.54) | 194.60(6.80) | 216.16(6.77) | 229.79(8.60) | < 0.0001 |
| Alcohol intake(g) | 5.93(0.51) | 12.04(1.16) | 14.15(0.80) | 16.43(1.10) | < 0.0001 |
| Na intake(mg) | 3485.73(38.85) | 3697.99(54.75) | 3607.19(40.34) | 3457.07(41.71) | < 0.001 |
| Cotinine (ng/ml) | 41.09(3.06) | 54.49(4.19) | 69.51(4.21) | 82.29(5.11) | < 0.0001 |

**Supplementary Table 1.** Clinical characteristics of the study population by Hcy quartiles.
