# Supplementary material for: Association between homocysteine levels and hypertension prevalence as well as all-cause mortality and cardiovascular mortality among hypertensive patients: A population-based study
Source: PLoS One. 2025 Aug 12;20(8):e0330267. doi: 10.1371/journal.pone.0330267 (PMC12342259; doi:10.1371/journal.pone.0330267)
Supplement: S1 File — (DOCX) [file pone.0330267.s002.docx]

library(nhanesR)

library(do)

library(dplyr)

library(reshape2)

library(survey)

library(openxlsx)

demo1 <- nhs_tsv('demo\\.',years = 1999)

d1_1 <- nhs_read(demo1,'ridageyr:age','riagendr:sex',

'ridreth1:reth1','dmdmartl:marriage',

'dmdeduc2:education','indfmpir:pir','sdmvpsu','sdmvstra','wtmec4yr',codebook = TRUE)

demo2 <- nhs_tsv('demo_b')

d1_2 <- nhs_read(demo2,'ridageyr:age','riagendr:sex',

'ridreth1:reth1','dmdmartl:marriage',

'dmdeduc2:education','indfmpir:pir','sdmvpsu','sdmvstra','wtmec4yr',codebook = TRUE)

demo3 <- nhs_tsv('demo_c|demo_d')

d1_3 <- nhs_read(demo3,'ridageyr:age','riagendr:sex',

'ridreth1:reth1','dmdmartl:marriage',

'dmdeduc2:education','indfmpir:pir','wtmec2yr:wtmec4yr',

'sdmvpsu','sdmvstra',codebook = TRUE,Year = T)

d1 <- rbind(d1_1,d1_2,d1_3)

hcy1 <- nhs_tsv('lab06')

d2_1 <- nhs_read(hcy1,'lbxhcy:Hcy',codebook = TRUE,Year = F)

hcy2 <- nhs_tsv('l06_b')

d2_2 <- nhs_read(hcy2,'lbdhcy:Hcy',codebook = TRUE,Year = F)

hcy3 <- nhs_tsv('l06mh_c|hcy_d')

d2_3 <- nhs_read(hcy3,'lbxhcy:Hcy',codebook = TRUE,Year = F)

d2 <- rbind(d2_1,d2_2,d2_3)

d3_1 <- dex_PhysicalActivity(years = 1999,all.5 = T,time = T,MET = T,Year = T)

d3_2 <- dex_PhysicalActivity(years = 2001,all.5 = T,time = T,MET = T,Year = T)

d3_3 <- dex_PhysicalActivity(years = 2003,all.5 = T,time = T,MET = T,Year = T)

d3_4 <- dex_PhysicalActivity(years = 2005,all.5 = T,time = T,MET = T,Year = T)

d3_5 <- rbind(d3_1,d3_2,d3_3,d3_4)

d3 <- drop_col(d3_5,'PA_total_time')

dr1tot1_0<- nhs_tsv('drxtot\\.',years = 1999)

d4_0 <- nhs_read(dr1tot1_0,'drxtcaff:caff',Year = FALSE)

dr1tot1<- nhs_tsv('drxtot_b')

d4_1 <- nhs_read(dr1tot1,'drxtcaff:caff',Year = FALSE)

dr1tot2<- nhs_tsv('dr1tot_c|dr1tot_d')

d4_2 <- nhs_read(dr1tot2,'dr1tcaff:caff1',Year = FALSE)

dr2tot3<- nhs_tsv('dr2tot_c|dr2tot_d')

d4_3 <- nhs_read(dr2tot3,'dr2tcaff:caff2',Year = FALSE)

d4_4 <- select_row(d4_2,d4_2$caff1 !='NA')

d4_5 <- Inner_Join(d4_3,d4_4)

d4_6 <- d4_5 %>%

mutate(caff = if_else(!is.na(caff1) & !is.na(caff2), (caff1 + caff2) / 2,

caff1))

d4_7 <- drop_col(d4_6,'caff1','caff2')

d4_8 <- select_row(d4_0,d4_0$caff !='NA')

d4_9 <- select_row(d4_1,d4_1$caff !='NA')

d4 <- rbind(d4_8,d4_7)

dr1tot4_0<- nhs_tsv('drxtot\\.',years = 1999)

d5_0 <- nhs_read(dr1tot4_0,'drxtalco:alco',Year = FALSE)

dr1tot4<- nhs_tsv('drxtot_b')

d5_1 <- nhs_read(dr1tot4,'drxtalco:alco',Year = FALSE)

dr1tot5<- nhs_tsv('dr1tot_c|dr1tot_d')

d5_2 <- nhs_read(dr1tot5,'dr1talco:alco1',Year = FALSE)

dr2tot6<- nhs_tsv('dr2tot_c|dr2tot_d')

d5_3 <- nhs_read(dr2tot6,'dr2talco:alco2',Year = FALSE)

d5_4 <- select_row(d5_2,d5_2$alco1 !='NA')

d5_5 <- Inner_Join(d5_3,d5_4)

d5_6 <- d5_5 %>%

mutate(alco = if_else(!is.na(alco1) & !is.na(alco2), (alco1 + alco2) / 2,

alco1))

d5_7 <- drop_col(d5_6,'alco1','alco2')

d5_8 <- select_row(d5_0,d5_0$alco !='NA')

d5_9 <- select_row(d5_1,d5_1$alco !='NA')

d5 <- rbind(d5_7,d5_8,d5_9)

na0 <- nhs_tsv('drxtot\\.',years = 1999)

d6_0 <- nhs_read(na0,'drdtsodi:Na',Year = FALSE)

na2<- nhs_tsv('drxtot_b')

d6_1 <- nhs_read(na2,'drdtsodi:Na',Year = FALSE)

dr1tot7<- nhs_tsv('dr1tot_c|dr1tot_d')

d6_2 <- nhs_read(dr1tot7,'dr1tsodi:Na1',Year = FALSE)

dr2tot8<- nhs_tsv('dr2tot_c|dr2tot_d')

d6_3 <- nhs_read(dr2tot8,'dr2tsodi:Na2',Year = FALSE)

d6_4 <- select_row(d6_2,d6_2$Na1 !='NA')

d6_5 <- Inner_Join(d6_3,d6_4)

d6_6 <- d6_5 %>%

mutate(Na = if_else(!is.na(Na1) & !is.na(Na2), (Na1 + Na2) / 2,

Na1))

d6_7 <- select_row(d6_0,d6_0$Na !='NA')

d6_8 <- select_row(d6_1,d6_1$Na !='NA')

d6_9 <- drop_col(d6_6,'Na1','Na2')

d6 <- rbind(d6_7,d6_8,d6_9)

Cotinine<- nhs_tsv('lab06|l06_b|l06cot_c|cot_d')

d7 <- nhs_read(Cotinine,'lbxcot:Cotinine',Year = FALSE)

bmx1 <- nhs_tsv('bmx\\.',years = 1999)

d8_0 <- nhs_read(bmx1, 'bmxbmi:bmi',

Year = FALSE)

bmx2 <- nhs_tsv('bmx_b|bmx_c|bmx_d')

d8_1 <- nhs_read(bmx2,'bmxbmi:bmi',Year = FALSE)

d8 <- rbind(d8_0,d8_1)

HF1 <- nhs_tsv('mcq\\.',years = 1999)

d9_0 <- nhs_read(HF1,'mcq160b:HF','mcq160c:CORD','MCQ160d:ANGD',

'MCQ160E:INFD','MCQ160F:STROKE',codebook = TRUE,Year = FALSE)

HF2 <- nhs_tsv('mcq_b|mcq_c|mcq_d')

d9_1 <- nhs_read(HF2,'mcq160b:HF','mcq160c:CORD','MCQ160d:ANGD',

'MCQ160E:INFD','MCQ160F:STROKE',Year = FALSE)

d9 <- rbind(d9_0,d9_1)

d10 <- diag_Hyperlipidemia(years = 1999:2006)

daibetes1 <- nhs_tsv('diq\\.',years = 1999)

d11_0 <- nhs_read(daibetes1,'DIQ010:diabetes',codebook = TRUE,Year = FALSE)

daibetes2 <- nhs_tsv('diq_b|diq_c|diq_d')

d11_1 <- nhs_read(daibetes2,'DIQ010:diabetes',codebook = TRUE,Year = FALSE)

d11 <- rbind(d11_0,d11_1)

d12 <- diag_Hypertension(years = 1999:2006)

death <- db_mort(years = 1999:2006,varLabel = F)

d13_0 <- select_col(death,'seqn','permth_exm','eligstat','mortstat',

'ucod_leading',Year = FALSE)

d13 <- select_row(d13_0,d13_0$eligstat =='Eligible')

table(d13$mortstat,useNA = 'i')

col_rename(d13) <- c('mortstat:status','permth_exm:time','ucod_leading:leading')

d13 <- add_col(data = d13,colname = 'leading',value = '0',condition = is.na(d13$leading))

d1 <- d1 %>% mutate(seqn = as.character(seqn))

d2 <- d2 %>% mutate(seqn = as.character(seqn))

d3 <- d3 %>% mutate(seqn = as.character(seqn))

d4 <- d4 %>% mutate(seqn = as.character(seqn))

d5 <- d5 %>% mutate(seqn = as.character(seqn))

d6 <- d6 %>% mutate(seqn = as.character(seqn))

d7 <- d7 %>% mutate(seqn = as.character(seqn))

d8 <- d8 %>% mutate(seqn = as.character(seqn))

d9 <- d9 %>% mutate(seqn = as.character(seqn))

d10 <- d10 %>% mutate(seqn = as.character(seqn))

d11 <- d11 %>% mutate(seqn = as.character(seqn))

d12 <- d12 %>% mutate(seqn = as.character(seqn))

d13 <- d13 %>% mutate(seqn = as.character(seqn))

d14 <- Full_Join(d1,d2,d3,d4,d5,d6,d7,d8,d9,d10,d11,d12,d13)

d14_8 <- select_row(d14,d14$Hypertension !='NA')

d15_1 <- select_row(d14_8,d14_8$age !='NA')

d15_2 <- select_row(d15_1,d15_1$sex !='NA')

d15_3 <- select_row(d15_2,d15_2$marriage !='NA')

d15_4 <- select_row(d15_3,d15_3$education !='NA')

d15_5 <- select_row(d15_4,d15_4$pir !='NA')

d15_6 <- select_row(d15_5,d15_5$reth1 !='NA')

d15_7 <- select_row(d15_6,d15_6$Hcy !='NA')

d15_8 <- select_row(d15_7,d15_7$bmi !='NA')

d16_0 <- select_row(d15_8,d15_8$diabetes !='NA')

d16_1 <- select_row(d16_0,d16_0$Cotinine !='NA')

d16_2 <- select_row(d16_1,d16_1$PA_total_MET !='NA')

d16_3 <- select_row(d16_2,d16_2$Na!='NA')

d16_4 <- select_row(d16_3,d16_3$alco!='NA')

d17_0 <- select_row(d16_4,d16_4$HF =='Yes'|d16_4$HF =='No')

d17_1 <- select_row(d17_0,d17_0$CORD =='Yes'|d17_0$CORD =='No')

d17_2 <- select_row(d17_1,d17_1$ANGD =='Yes'|d17_1$ANGD =='No')

d17_3 <- select_row(d17_2,d17_2$INFD =='Yes'|d17_2$INFD =='No')

Total <- select_row(d17_3,d17_3$STROKE =='Yes'|d17_3$STROKE =='No')

mmhg1 <- nhs_tsv('bpx\\.',years = 1999)

d12_0 <- nhs_read(mmhg1,'bpxsy1:S1','bpxsy2:S2','bpxsy3:S3',

'bpxdi1:D1','bpxdi2:D2','bpxdi3:D3',codebook = TRUE,Year = FALSE)

mmhg2 <- nhs_tsv('bpx_b|bpx_c|bpx_d')

d12_1 <- nhs_read(mmhg2,'bpxsy1:S1','bpxsy2:S2','bpxsy3:S3',

'bpxdi1:D1','bpxdi2:D2','bpxdi3:D3',codebook = TRUE,Year = FALSE)

d12 <- rbind(d12_0,d12_1)

Total <- Inner_Join(Total,d12)

demo1 <- nhs_tsv('demo\\.',years = 1999)

d1_1 <- nhs_read(demo1,'ridageyr:age','riagendr:sex',

'ridreth1:reth1','dmdmartl:marriage',

'dmdeduc2:education','indfmpir:pir','sdmvpsu','sdmvstra','wtmec4yr',codebook = TRUE)

demo2 <- nhs_tsv('demo_b')

d1_2 <- nhs_read(demo2,'ridageyr:age','riagendr:sex',

'ridreth1:reth1','dmdmartl:marriage',

'dmdeduc2:education','indfmpir:pir','sdmvpsu','sdmvstra','wtmec4yr',codebook = TRUE)

demo3 <- nhs_tsv('demo_c|demo_d')

d1_3 <- nhs_read(demo3,'ridageyr:age','riagendr:sex',

'ridreth1:reth1','dmdmartl:marriage',

'dmdeduc2:education','indfmpir:pir','wtmec2yr:wtmec4yr',

'sdmvpsu','sdmvstra',codebook = TRUE,Year = T)

d1 <- rbind(d1_1,d1_2,d1_3)

nrow(d1)

hcy1 <- nhs_tsv('lab06')

d2_1 <- nhs_read(hcy1,'lbxhcy:Hcy',codebook = TRUE,Year = F)

hcy2 <- nhs_tsv('l06_b')

d2_2 <- nhs_read(hcy2,'lbdhcy:Hcy',codebook = TRUE,Year = F)

hcy3 <- nhs_tsv('l06mh_c|hcy_d')

d2_3 <- nhs_read(hcy3,'lbxhcy:Hcy',codebook = TRUE,Year = F)

d2 <- rbind(d2_1,d2_2,d2_3)

d22 <- full_join(d1,d2)

d12 <- diag_Hypertension(years = 1999:2006)

d11 <- full_join(d22,d12)

death <- db_mort(years = 1999:2006,varLabel = F)

d133 <- select_col(death,'seqn','permth_exm','eligstat','mortstat',

'ucod_leading',Year = FALSE)

col_rename(d133) <- c('mortstat:status','permth_exm:time','ucod_leading:leading')

d1333 <- add_col(data = d133,colname = 'leading',value = '0',condition = is.na(d133$leading))

d11 <- d11 %>% mutate(seqn = as.character(seqn))

d1333 <- d1333 %>% mutate(seqn = as.character(seqn))

d13333 <- full_join(d11,d1333)

d222 <- select_row(d13333,d13333$age>=18)

d2222 <- select_row(d222,d222$Hcy !='NA')

d111 <- select_row(d2222,d2222$Hypertension !='NA')

d133333 <- select_row(d111,d111$eligstat =='Eligible')

d1333333 <- select_row(d133333,d133333$status!='NA')

d3_1 <- dex_PhysicalActivity(years = 1999,all.5 = T,time = T,MET = T,Year = T)

d3_2 <- dex_PhysicalActivity(years = 2001,all.5 = T,time = T,MET = T,Year = T)

d3_3 <- dex_PhysicalActivity(years = 2003,all.5 = T,time = T,MET = T,Year = T)

d3_4 <- dex_PhysicalActivity(years = 2005,all.5 = T,time = T,MET = T,Year = T)

d3_5 <- rbind(d3_1,d3_2,d3_3,d3_4)

d3 <- drop_col(d3_5,'PA_total_time')

dr1tot1_0<- nhs_tsv('drxtot\\.',years = 1999)

d4_0 <- nhs_read(dr1tot1_0,'drxtcaff:caff',Year = FALSE)

dr1tot1<- nhs_tsv('drxtot_b')

d4_1 <- nhs_read(dr1tot1,'drxtcaff:caff',Year = FALSE)

dr1tot2<- nhs_tsv('dr1tot_c|dr1tot_d')

d4_2 <- nhs_read(dr1tot2,'dr1tcaff:caff1',Year = FALSE)

dr2tot3<- nhs_tsv('dr2tot_c|dr2tot_d')

d4_3 <- nhs_read(dr2tot3,'dr2tcaff:caff2',Year = FALSE)

d4_4 <- select_row(d4_2,d4_2$caff1 !='NA')

d4_5 <- Inner_Join(d4_3,d4_4)

d4_6 <- d4_5 %>%

mutate(caff = if_else(!is.na(caff1) & !is.na(caff2), (caff1 + caff2) / 2,

caff1))

d4_7 <- drop_col(d4_6,'caff1','caff2')

d4_8 <- select_row(d4_0,d4_0$caff !='NA')

d4_9 <- select_row(d4_1,d4_1$caff !='NA')

d4 <- rbind(d4_7,d4_8,d4_9)

dr1tot4_0<- nhs_tsv('drxtot\\.',years = 1999)

d5_0 <- nhs_read(dr1tot4_0,'drxtalco:alco',Year = FALSE)

dr1tot4<- nhs_tsv('drxtot_b')

d5_1 <- nhs_read(dr1tot4,'drxtalco:alco',Year = FALSE)

dr1tot5<- nhs_tsv('dr1tot_c|dr1tot_d')

d5_2 <- nhs_read(dr1tot5,'dr1talco:alco1',Year = FALSE)

dr2tot6<- nhs_tsv('dr2tot_c|dr2tot_d')

d5_3 <- nhs_read(dr2tot6,'dr2talco:alco2',Year = FALSE)

d5_4 <- select_row(d5_2,d5_2$alco1 !='NA')

d5_5 <- Inner_Join(d5_3,d5_4)

d5_6 <- d5_5 %>%

mutate(alco = if_else(!is.na(alco1) & !is.na(alco2), (alco1 + alco2) / 2,

alco1))

d5_7 <- drop_col(d5_6,'alco1','alco2')

d5_8 <- select_row(d5_0,d5_0$alco !='NA')

d5_9 <- select_row(d5_1,d5_1$alco !='NA')

d5 <- rbind(d5_7,d5_8,d5_9)

na0 <- nhs_tsv('drxtot\\.',years = 1999)

d6_0 <- nhs_read(na0,'drdtsodi:Na',Year = FALSE)

na2<- nhs_tsv('drxtot_b')

d6_1 <- nhs_read(na2,'drdtsodi:Na',Year = FALSE)

dr1tot7<- nhs_tsv('dr1tot_c|dr1tot_d')

d6_2 <- nhs_read(dr1tot7,'dr1tsodi:Na1',Year = FALSE)

dr2tot8<- nhs_tsv('dr2tot_c|dr2tot_d')

d6_3 <- nhs_read(dr2tot8,'dr2tsodi:Na2',Year = FALSE)

d6_4 <- select_row(d6_2,d6_2$Na1 !='NA')

d6_5 <- Inner_Join(d6_3,d6_4)

d6_6 <- d6_5 %>%

mutate(Na = if_else(!is.na(Na1) & !is.na(Na2), (Na1 + Na2) / 2,

Na1))

d6_7 <- select_row(d6_0,d6_0$Na !='NA')

d6_8 <- select_row(d6_1,d6_1$Na !='NA')

d6_9 <- drop_col(d6_6,'Na1','Na2')

d6 <- rbind(d6_7,d6_8,d6_9)

Cotinine<- nhs_tsv('lab06|l06_b|l06cot_c|cot_d')

d7 <- nhs_read(Cotinine,'lbxcot:Cotinine',Year = FALSE)

bmx1 <- nhs_tsv('bmx\\.',years = 1999)

d8_0 <- nhs_read(bmx1, 'bmxbmi:bmi',

Year = FALSE)

bmx2 <- nhs_tsv('bmx_b|bmx_c|bmx_d')

d8_1 <- nhs_read(bmx2,'bmxbmi:bmi',Year = FALSE)

d8 <- rbind(d8_0,d8_1)

nrow(d8)

HF1 <- nhs_tsv('mcq\\.',years = 1999)

d9_0 <- nhs_read(HF1,'mcq160b:HF','mcq160c:CORD','MCQ160d:ANGD',

'MCQ160E:INFD','MCQ160F:STROKE',codebook = TRUE,Year = FALSE)

HF2 <- nhs_tsv('mcq_b|mcq_c|mcq_d')

d9_1 <- nhs_read(HF2,'mcq160b:HF','mcq160c:CORD','MCQ160d:ANGD',

'MCQ160E:INFD','MCQ160F:STROKE',Year = FALSE)

d9 <- rbind(d9_0,d9_1)

d10 <- diag_Hyperlipidemia(years = 1999:2006)

daibetes1 <- nhs_tsv('diq\\.',years = 1999)

d11_0 <- nhs_read(daibetes1,'DIQ010:diabetes',codebook = TRUE,Year = FALSE)

daibetes2 <- nhs_tsv('diq_b|diq_c|diq_d')

d11_1 <- nhs_read(daibetes2,'DIQ010:diabetes',codebook = TRUE,Year = FALSE)

d11 <- rbind(d11_0,d11_1)

mmhg1 <- nhs_tsv('bpx\\.',years = 1999)

d12_0 <- nhs_read(mmhg1,'bpxsy1:S1','bpxsy2:S2','bpxsy3:S3',

'bpxdi1:D1','bpxdi2:D2','bpxdi3:D3',codebook = TRUE,Year = FALSE)

mmhg2 <- nhs_tsv('bpx_b|bpx_c|bpx_d')

d12_1 <- nhs_read(mmhg2,'bpxsy1:S1','bpxsy2:S2','bpxsy3:S3',

'bpxdi1:D1','bpxdi2:D2','bpxdi3:D3',codebook = TRUE,Year = FALSE)

d12 <- rbind(d12_0,d12_1)

d1333333 <- d1333333 %>% mutate(seqn = as.character(seqn))

d3 <- d3 %>% mutate(seqn = as.character(seqn))

d4 <- d4 %>% mutate(seqn = as.character(seqn))

d5 <- d5 %>% mutate(seqn = as.character(seqn))

d6 <- d6 %>% mutate(seqn = as.character(seqn))

d7 <- d7 %>% mutate(seqn = as.character(seqn))

d8 <- d8 %>% mutate(seqn = as.character(seqn))

d9 <- d9 %>% mutate(seqn = as.character(seqn))

d10 <- d10 %>% mutate(seqn = as.character(seqn))

d11 <- d11 %>% mutate(seqn = as.character(seqn))

d12 <- d12 %>% mutate(seqn = as.character(seqn))

d14 <- full_join(d1333333,d3)

d14_0 <- full_join(d14,d4)

d14_1 <- full_join(d14_0,d5)

d14_2 <- full_join(d14_1,d6)

d14_3 <- full_join(d14_2,d7)

d14_4 <- full_join(d14_3,d8)

d14_5 <- full_join(d14_4,d9)

d14_6 <- full_join(d14_5,d10)

d14_7 <- full_join(d14_6,d11)

d14_8 <- full_join(d14_7,d12)

d15_1 <- select_row(d14_8,d14_8$age !='NA')

d15_2 <- select_row(d15_1,d15_1$sex !='NA')

d15_3 <- select_row(d15_2,d15_2$marriage !='NA')

d15_4 <- select_row(d15_3,d15_3$education !='NA')

d15_5 <- select_row(d15_4,d15_4$pir !='NA')

d15_6 <- select_row(d15_5,d15_5$reth1 !='NA')

d15_8 <- select_row(d15_6,d15_6$bmi !='NA')

d16_0 <- select_row(d15_8,d15_8$diabetes !='NA')

d16_1 <- select_row(d16_0,d16_0$Cotinine !='NA')

d16_2 <- select_row(d16_1,d16_1$PA_total_MET !='NA')

d16_3 <- select_row(d16_2,d16_2$Na!='NA')

d16_4 <- select_row(d16_3,d16_3$alco!='NA')

d17_0 <- select_row(d16_4,d16_4$HF =='Yes'|d16_4$HF =='No')

d17_1 <- select_row(d17_0,d17_0$CORD =='Yes'|d17_0$CORD =='No')

d17_2 <- select_row(d17_1,d17_1$ANGD =='Yes'|d17_1$ANGD =='No')

d17_3 <- select_row(d17_2,d17_2$INFD =='Yes'|d17_2$INFD =='No')

d17_4 <- select_row(d17_3,d17_3$STROKE =='Yes'|d17_3$STROKE =='No')

d17_5 <- select_row(d17_4,d17_4$D3 !='NA')

d17_6 <- select_row(d17_5,d17_5$S1 !='NA')

d17_7 <- select_row(d17_6,d17_6$S2 !='NA')

d17_8 <- select_row(d17_7,d17_7$S3 !='NA')

d17_9 <- select_row(d17_8,d17_8$D1 !='NA')

Total <- select_row(d17_9,d17_9$D2 !='NA')

Total$ST <- 1/3*Total$S1+1/3*Total$S2+1/3*Total$S3

Total$DT <- 1/3*Total$D1+1/3*Total$D2+1/3*Total$D3

Total <- drop_col(Total,'S1', 'S2', 'S3', 'D1', 'D2', 'D3')

Total1 <- select_row(Total,Total$status!='NA')

table(Total1$Hypertension)

missValue(Total1)

Total2 <- select_row(Total1,Total1$Hypertension!='no')

missValue(Total1)

missValue(Total2)

table(Total2$status)

# Recode(Total$HF)

Total1$HF <- Recode(Total1$HF,

"No::0",

"Yes::1",

to.numeric = T)

# Recode(Total$CORD)

Total1$CORD <- Recode(Total1$CORD,

"No::0",

"Yes::1",

to.numeric = T)

# Recode(Total$ANGD)

Total1$ANGD <- Recode(Total1$ANGD,

"No::0",

"Yes::1",

to.numeric = T)

# Recode(Total$INFD)

Total1$INFD <- Recode(Total1$INFD,

"No::0",

"Yes::1",

to.numeric = T)

# Recode(Total$STROKE)

Total1$STROKE <- Recode(Total1$STROKE,

"No::0",

"Yes::1",

to.numeric = T)

Total1$CVD <- ifelse(rowSums(Total1[, c("HF", "CORD", "ANGD", "INFD","STROKE")] == 1) > 0, 'Yes', 'No')

Total1 <- drop_col(Total1,'HF','CORD','ANGD','INFD','STROKE')

missValue(Total1)

Total1$pir1 <- Total1$pir

bu_x<- Total1$pir1

Total1$pir1[bu('( , 1)')]<-'<1'

Total1$pir1[bu('[1 , 3]')]<-'1-3'

Total1$pir1[bu('(3 , )')]<-'>3'

# Recode(Total1$reth1)

Total1$reth1 <- Recode(Total1$reth1,

"Non-Hispanic Black::",

"Non-Hispanic White::",

"Mexican American::",

"Other Hispanic::Other Race",

"Other Race - Including Multi-Racial::Other Race",

to.numeric = FALSE)

# Recode(Total1$marriage)

Total1$marriage <- Recode(Total1$marriage,

"Never married::single",

"Living with partner::",

"Married::",

"Divorced::single",

"Widowed::single",

"Separated::single",

to.numeric = FALSE)

# Recode(Total1$education)

Total1$education <- Recode(Total1$education,

"9-11th Grade (Includes 12th grade with no diploma)::Less than high school",

"High School Grad/GED or Equivalent::High school",

"Some College or AA degree::More than high school",

"Less Than 9th Grade::Less than high school",

"College Graduate or above::More than high school",

"High school graduate/GED or equivalent::High school",

"Some college or AA degree::More than high school",

"College graduate or above::More than high school",

"9-11th grade (Includes 12th grade with no diploma)::Less than high school",

"Less than 9th grade::Less than high school",

to.numeric = FALSE)

# Factor(Total1$marriage)

Total1$marriage <- factor(Total1$marriage, levels = c("single","Living with partner","Married"))

# Factor(Total$education)

Total1$education <- factor(Total1$education, levels = c("Less than high school","High school","More than high school"))

# Factor(Total1$diabetes)

Total1$diabetes <- factor(Total1$diabetes, levels = c("No","Borderline","Yes"))

# Factor(Total$pir)

Total1$pir1 <- factor(Total1$pir1, levels = c("<1","1-3",">3"))

# Recode(Total2$sarcopenia_obesity)

Total1$Hypertension <- Recode(Total1$Hypertension,

"no::0",

"yes::1",

to.numeric = T)

table(Total1$Hypertension)

# Recode(Total1$status)

Total1$status <- Recode(Total1$status,

"Assumed alive::0",

"Assumed deceased::1",

to.numeric = T)

#计算权重

# 合并年份

ck <- Total$Year %in% c('1999-2000','2001-2002')

Total$nhs_wt <- ifelse(ck,2/4*Total$wtmec4yr,1/4*Total$wtmec4yr)

Total <- drop_row(Total,Total$nhs_wt == '0')

Total2 <- select_row(Total1,Total1$Hypertension !='0')

Total2$leading_CVD <- Total2$leading

# Recode(Total$leading_CVD)

Total2$leading_CVD <- Recode(Total2$leading_CVD,

"0::",

"Diseases of heart (I00-I09, I11, I13, I20-I51)::1",

"All other causes (residual)::2",

"Malignant neoplasms (C00-C97)::2",

"Cerebrovascular diseases (I60-I69)::1",

"Chronic lower respiratory diseases (J40-J47)::2",

"Nephritis, nephrotic syndrome and nephrosis (N00-N07, N17-N19, N25-N27)::2",

"Alzheimer's disease (G30)::2",

"Accidents (unintentional injuries) (V01-X59, Y85-Y86)::2",

"Influenza and pneumonia (J09-J18)::2",

"Diabetes mellitus (E10-E14)::2",

to.numeric = T)

Total3 <- select_row(Total2,Total2$leading_CVD !='2')

setwd('D:\\Desktop\\BUN Sarcopenia\\Homocysteinehypertention')

getwd()

write.csv(Total1,file = "total1.csv",row.names = F)

write.csv(Total2,file = "total2.csv",row.names = F)

write.csv(Total3,file = "total3.csv",row.names = F)

Total1 <- read.csv("total1.csv")

Total2 <- read.csv("total2.csv")

Total3 <- read.csv("total3.csv")

table(Total2$status)

table(Total3$leading_CVD)

Total1_nhs <- svy_design(Total1)

Total2_nhs <- svy_design(Total2)

Total3_nhs <- svy_design(Total3)

dd1 <- svy_tableone(design = Total1_nhs,cv = c('age','PA_total_MET','caff','alco','Na',

'Cotinine','bmi','Hcy','ST','DT'

),total = T,

by = 'Hypertension')

dd2 <- svy_tableone(design = Total1_nhs, gv = c('sex','reth1','marriage','education',

'pir1','Hyperlipidemia','CVD',

'diabetes'),total = T,

g_perSQse = TRUE,by = 'Hypertension')

# quant(Total1$Hcy)

Total1$HcyQ <- quant(Total1$Hcy, n = 4,Q = TRUE,round=3)

Total1$HcyQ.median <- quant.median(Total1$Hcy, n =4 ,round=3)

Total1_nhs <- svy_design(Total1)

dd3 <- svy_tableone(design = Total1_nhs,cv = c('age','PA_total_MET','caff','alco','Na',

'Cotinine','bmi','ST','DT'

),total = T,

by = 'HcyQ')

dd4 <- svy_tableone(design = Total1_nhs, gv = c('sex','reth1','marriage','education',

'pir1','Hyperlipidemia','CVD',

'diabetes','Hypertension'),total = T,

g_perSQse = TRUE,by = 'HcyQ')

dd3_0 <- svyglm( Hypertension ~ Hcy,Total1_nhs,family = quasibinomial) %>% reg_table()

dd3_1 <- svyglm( Hypertension ~ HcyQ,Total1_nhs,family = quasibinomial) %>% reg_table()

dd3_2 <- svyglm( Hypertension ~ Hcy+age+sex+reth1+education,Total1_nhs,family = quasibinomial) %>% reg_table()

dd3_3 <- svyglm( Hypertension ~ HcyQ+age+sex+reth1+education,Total1_nhs,family = quasibinomial) %>% reg_table()

dd3_4 <- svyglm( Hypertension ~ Hcy+age+sex+reth1+education+Na+Cotinine+bmi+pir1+Hyperlipidemia+CVD+diabetes,Total1_nhs,family = quasibinomial) %>% reg_table()

dd3_5 <- svyglm( Hypertension ~ HcyQ+age+sex+reth1+education+Na+Cotinine+bmi+pir1+Hyperlipidemia+CVD+diabetes,Total1_nhs,family = quasibinomial) %>% reg_table()

p4trend(dd3_1,'HcyQ')

p4trend(dd3_3,'HcyQ')

p4trend(dd3_5,'HcyQ')

# quant(Total1$Hcy)

Total1$HcyQ <- quant(Total1$Hcy, n = 4,Q = TRUE,round=3)

Total1$HcyQ.median <- quant.median(Total1$Hcy, n =4 ,round=3)

Total1_nhs <- svy_design(Total1)

dd4_0 <- svyglm( ST ~ Hcy,Total1_nhs) %>% reg_table()

dd4_1 <- svyglm( ST ~ HcyQ,Total1_nhs) %>% reg_table()

dd4_2 <- svyglm( ST ~ Hcy+age+sex+reth1+education,Total1_nhs) %>% reg_table()

dd4_3 <- svyglm( ST ~ HcyQ+age+sex+reth1+education,Total1_nhs) %>% reg_table()

dd4_4 <- svyglm( ST ~ Hcy+age+sex+reth1+education+Na+Cotinine+bmi+pir1+Hyperlipidemia+CVD+diabetes,Total1_nhs) %>% reg_table()

dd4_5 <- svyglm( ST ~ HcyQ+age+sex+reth1+education+Na+Cotinine+bmi+pir1+Hyperlipidemia+CVD+diabetes,Total1_nhs) %>% reg_table()

dd5_0 <- svyglm( DT ~ Hcy,Total1_nhs) %>% reg_table()

dd5_1 <- svyglm( DT ~ HcyQ,Total1_nhs) %>% reg_table()

dd5_2 <- svyglm( DT ~ Hcy+age+sex+reth1+education,Total1_nhs) %>% reg_table()

dd5_3 <- svyglm( DT ~ HcyQ+age+sex+reth1+education,Total1_nhs) %>% reg_table()

dd5_4 <- svyglm( DT ~ Hcy+age+sex+reth1+education+Na+Cotinine+bmi+pir1+Hyperlipidemia+CVD+diabetes,Total1_nhs) %>% reg_table()

dd5_5 <- svyglm( DT ~ HcyQ+age+sex+reth1+education+Na+Cotinine+bmi+pir1+Hyperlipidemia+CVD+diabetes,Total1_nhs) %>% reg_table()

p4trend(dd4_1,'HcyQ')

p4trend(dd4_3,'HcyQ')

p4trend(dd4_5,'HcyQ')

p4trend(dd5_1,'HcyQ')

p4trend(dd5_3,'HcyQ')

p4trend(dd5_5,'HcyQ')

library(rms)

#RCS

library(rms)

f0 <- svyglm( ST ~ rcs(Hcy,3)+age+sex+reth1+education+Na+Cotinine+bmi+pir1+Hyperlipidemia+CVD+diabetes,Total1_nhs)

dd6 <- optimal_nKnots(f0)

r0 <- RCS(f0,log = F)

ggplot(r0)

getChangepoints(r0)

f1 <- svyglm( DT ~ rcs(Hcy,3)+age+sex+reth1+education+Na+Cotinine+bmi+pir1+Hyperlipidemia+CVD+diabetes,Total1_nhs)

# f1 <- lm( DT ~ rcs(Hcy,4)+age+sex+reth1+education+Na+Cotinine+bmi+pir1+Hyperlipidemia+CVD+diabetes,Total1)

dd6 <- optimal_nKnots(f1)

r1 <- RCS(f1,log = F)

ggplot(r1)

getChangepoints(r1)

Total1$Hcy1 <- Total1$Hcy

bu_x<- Total1$Hcy1

Total1$Hcy1[bu('( , 7.65)')]<-'D1'

Total1$Hcy1[bu('[7.65, )')]<-'D2'

table(Total1$Hcy1)

Total1_nhs <- svy_design(Total1)

Total11 <- select_row(Total1,Total1$Hcy1 =='D1')

Total11_nhs <- svy_design(Total11)

dd4_0 <- svyglm( DT ~ Hcy,Total11_nhs) %>% reg_table()

Total12 <- select_row(Total1,Total1$Hcy1 =='D2')

Total12_nhs <- svy_design(Total12)

dd4_1 <- svyglm( DT ~ Hcy,Total12_nhs) %>% reg_table()

Total1_nhs <- svy_design(Total1)

dd6 <- stratum_model(object = Total1_nhs,

y = 'DT',

x = 'Hcy',

stratum = c('Hcy1'),

adjust = c('age','sex','reth1','education','Na','Cotinine','bmi','pir1'

,'Hyperlipidemia','CVD','diabetes'))

write.xlsx(dd6,file = "dd6舒张压校正后分段线性回归.xlsx",rowNames = F)

library(segmented)

model_I <- svyglm( DT ~ Hcy

+age+sex+reth1+education+Na+Cotinine+bmi+pir1+Hyperlipidemia+CVD+diabetes,Total1_nhs)

attach(Total1_nhs)

model_II <- segmented(model_I, seg.Z = ~Hcy, psi = list(Hcy= 7.65))

test_result <- anova(model_I, model_II)

print(test_result)

if (test_result[2, "Pr(>F)"] < 0.05) {

cat("两模型具有统计学差异，拒绝单线模型。\n")

} else {

cat("两模型没有统计学差异，接受单线模型。\n")

}

model_I <- lm( DT ~ Hcy +age+sex+reth1+education+Na+Cotinine+bmi+pir1+Hyperlipidemia+CVD+diabetes,Total1)

Total1$Age<- Total1$age

bu_x<- Total1$Age

Total1$Age[bu('[18 , 60)')]<-'<60'

Total1$Age[bu('[60 , )')]<-'>=60'

Total1$BMI<- Total1$bmi

bu_x<- Total1$BMI

Total1$BMI[bu('( , 18.5)')]<-'Underweight'

Total1$BMI[bu('[18.5 , 24.9)')]<-'Normal weight'

Total1$BMI[bu('[24.9 , 29.9 )')]<-'Overweight'

Total1$BMI[bu('[29.9 , )')]<-'Obesity'

Total1$Sex<- Total1$sex

Total1$Race<- Total1$reth1

Total1$Pir<- Total1$pir1

Total1$Diabetes<- Total1$diabetes

# Recode(Total1$Hyperlipidemia)

Total1$Hyperlipidemia <- Recode(Total1$Hyperlipidemia,

"yes::Yes",

"no::No",

to.numeric = FALSE)

Total1_nhs <- svy_design(Total1)

f2 <- stratum_model(object = Total1_nhs,

y = 'Hypertension',

x = 'Hcy',

stratum = c('Age','BMI','Sex','Race','Pir',

'Diabetes','Hyperlipidemia','CVD'))

plot_df <- f2

col_rename(plot_df) <- c('95% CI:HR(95% CI)')

library(dplyr)

library(stringr)

library(purrr)

library(forestploter)

library(grid)

plot_df <- plot_df %>%

mutate(HR = "")

plot_df <- plot_df %>%

mutate(Lower = "")

plot_df <- plot_df %>%

mutate(Upper = "")

d1 <- plot_df <- plot_df %>%

mutate(

splitted_values = str_split_fixed(`HR(95% CI)`, "\\(|\\,|\\)", 3),

HR = ifelse(str_trim(splitted_values[, 1]) != "", str_trim(splitted_values[, 1]), " "),

Lower = ifelse(str_trim(splitted_values[, 2]) != "", str_trim(splitted_values[, 2]), " "),

Upper = ifelse(str_trim(splitted_values[, 3]) != "", str_remove_all(str_trim(splitted_values[, 3]), "\\)"), " ")

)

d2 <- select_col(d1,'character','HR(95% CI)','p','p for interaction','HR','Lower','Upper')

d2[d2 == " "] <- NA

d2$character <- ifelse(is.na(d2$HR),

d2$character,

paste0(" ", d2$character))

d2$` ` <- paste(rep(" ", nrow(d2)), collapse = " ")

kb <- paste(rep(" ", nrow(d2)), collapse = " ")

d3 <- cbind(d2$character,kb,d2[,2:4])

d2$HR <- as.numeric(as.character(d2$HR))

d2$Lower <- as.numeric(as.character(d2$Lower))

d2$Upper <- as.numeric(as.character(d2$Upper))

forest(d3[,c(1:5)],

est = d2$HR,

lower = d2$Lower, upper = d2$Upper,

sizes = 0.5, ci_column = 2, ref_line = 1,

xlim = c(0.6, 1.5),

ticks_at = c(0, 1, 2, 3,4,5,6))

Total1$Age<- Total1$age

bu_x<- Total1$Age

Total1$Age[bu('[18 , 60)')]<-'<60'

Total1$Age[bu('[60 , )')]<-'>=60'

Total1$BMI<- Total1$bmi

bu_x<- Total1$BMI

Total1$BMI[bu('( , 18.5)')]<-'Underweight'

Total1$BMI[bu('[18.5 , 24.9)')]<-'Normal weight'

Total1$BMI[bu('[24.9 , 29.9 )')]<-'Overweight'

Total1$BMI[bu('[29.9 , )')]<-'Obesity'

Total1$Sex<- Total1$sex

Total1$Race<- Total1$reth1

Total1$Pir<- Total1$pir1

Total1$Diabetes<- Total1$diabetes

Total1_nhs <- svy_design(Total1)

f3 <- stratum_model(object = Total1_nhs,

y = 'ST',

x = 'Hcy',

stratum = c('Age','BMI','Sex','Race','Pir',

'Diabetes','Hyperlipidemia','CVD'))

plot_df <- f3

col_rename(plot_df) <- c('95% CI:HR(95% CI)')

library(dplyr)

library(stringr)

library(purrr)

library(forestploter)

library(grid)

# install.packages(forestplot)

plot_df <- plot_df %>%

mutate(HR = "")

plot_df <- plot_df %>%

mutate(Lower = "")

plot_df <- plot_df %>%

mutate(Upper = "")

d1 <- plot_df <- plot_df %>%

mutate(

splitted_values = str_split_fixed(`HR(95% CI)`, "\\(|\\,|\\)", 3),

HR = ifelse(str_trim(splitted_values[, 1]) != "", str_trim(splitted_values[, 1]), " "),

Lower = ifelse(str_trim(splitted_values[, 2]) != "", str_trim(splitted_values[, 2]), " "),

Upper = ifelse(str_trim(splitted_values[, 3]) != "", str_remove_all(str_trim(splitted_values[, 3]), "\\)"), " ")

)

d2 <- select_col(d1,'character','HR(95% CI)','p','p for interaction','HR','Lower','Upper')

d2[d2 == " "] <- NA

d2$character <- ifelse(is.na(d2$HR),

d2$character,

paste0(" ", d2$character))

d2$` ` <- paste(rep(" ", nrow(d2)), collapse = " ")

kb <- paste(rep(" ", nrow(d2)), collapse = " ")

d3 <- cbind(d2$character,kb,d2[,2:4])

d2$HR <- as.numeric(as.character(d2$HR))

d2$Lower <- as.numeric(as.character(d2$Lower))

d2$Upper <- as.numeric(as.character(d2$Upper))

forest(d3[,c(1:5)],

est = d2$HR,

lower = d2$Lower,

upper = d2$Upper,

sizes = 0.5,

ci_column = 2,

ref_line = 0,

xlim = c(-1, 1.5),

ticks_at = c(0, 1, 2, 3,4,5,6))

Total1$Age<- Total1$age

bu_x<- Total1$Age

Total1$Age[bu('[18 , 60)')]<-'<60'

Total1$Age[bu('[60 , )')]<-'>=60'

Total1$BMI<- Total1$bmi

bu_x<- Total1$BMI

Total1$BMI[bu('( , 18.5)')]<-'Underweight'

Total1$BMI[bu('[18.5 , 24.9)')]<-'Normal weight'

Total1$BMI[bu('[24.9 , 29.9 )')]<-'Overweight'

Total1$BMI[bu('[29.9 , )')]<-'Obesity'

Total1$Sex<- Total1$sex

Total1$Race<- Total1$reth1

Total1$Pir<- Total1$pir1

Total1$Diabetes<- Total1$diabetes

Total1_nhs <- svy_design(Total1)

f3 <- stratum_model(object = Total1_nhs,

y = 'DT',

x = 'Hcy',

stratum = c('Age','BMI','Sex','Race','Pir',

'Diabetes','Hyperlipidemia','CVD'))

plot_df <- f3

col_rename(plot_df) <- c('95% CI:HR(95% CI)')

library(dplyr)

library(stringr)

library(purrr)

library(forestploter)

library(grid)

# install.packages(forestplot)

plot_df <- plot_df %>%

mutate(HR = "")

plot_df <- plot_df %>%

mutate(Lower = "")

plot_df <- plot_df %>%

mutate(Upper = "")

d1 <- plot_df <- plot_df %>%

mutate(

splitted_values = str_split_fixed(`HR(95% CI)`, "\\(|\\,|\\)", 3),

HR = ifelse(str_trim(splitted_values[, 1]) != "", str_trim(splitted_values[, 1]), " "),

Lower = ifelse(str_trim(splitted_values[, 2]) != "", str_trim(splitted_values[, 2]), " "),

Upper = ifelse(str_trim(splitted_values[, 3]) != "", str_remove_all(str_trim(splitted_values[, 3]), "\\)"), " ")

)

d2 <- select_col(d1,'character','HR(95% CI)','p','p for interaction','HR','Lower','Upper')

d2[d2 == " "] <- NA

d2$character <- ifelse(is.na(d2$HR),

d2$character,

paste0(" ", d2$character))

d2$` ` <- paste(rep(" ", nrow(d2)), collapse = " ")

kb <- paste(rep(" ", nrow(d2)), collapse = " ")

d3 <- cbind(d2$character,kb,d2[,2:4])

d2$HR <- as.numeric(as.character(d2$HR))

d2$Lower <- as.numeric(as.character(d2$Lower))

d2$Upper <- as.numeric(as.character(d2$Upper))

forest(d3[,c(1:5)],

est = d2$HR,

lower = d2$Lower,

upper = d2$Upper,

sizes = 0.5,

ci_column = 2,

ref_line = 0,

xlim = c(-1, 1.5),

ticks_at = c(0, 1, 2, 3,4,5,6))

Total2$Hcy1<- Total2$Hcy

bu_x<- Total2$Hcy1

Total2$Hcy1[bu('[10 , )')]<-'High'

Total2$Hcy1[bu('( , 10)')]<-'Low'

# Factor(Total2$Hcy1)

Total2$Hcy1 <- factor(Total2$Hcy1, levels = c("Low","High"))

Total2_nhs <- svy_design(Total2)

S1 <- svykm(Surv(time,status)~Hcy1,Total2_nhs)

# S1 <- svykm(Surv(time,status)~Hcy1, ci = T,Total2_nhs)

svy_kmplot(S1, ci = T,ylim = c(0,1))

Total3$Hcy1<- Total3$Hcy

bu_x<- Total3$Hcy1

Total3$Hcy1[bu('[10 , )')]<-'High'

Total3$Hcy1[bu('( , 10)')]<-'Low'

# Factor(Total3$Hcy1)

Total3$Hcy1 <- factor(Total3$Hcy1, levels = c("Low","High"))

Total3_nhs <- svy_design(Total3)

S2 <- svykm(Surv(time,status)~Hcy1,Total3_nhs)

svy_kmplot(S2, ci = T,ylim = c(0.5,1))

# quant(Total2$Hcy)

Total2$HcyQ <- quant(Total2$Hcy, n = 4,Q = TRUE,round=3)

Total2$HcyQ.median <- quant.median(Total2$Hcy, n = 4,round=3)

Total2_nhs <- svy_design(Total2)

#moldel1

f3 <- svycoxph(Surv(time,status) ~ Hcy1,Total2_nhs) %>% reg_table(round = 3)

f4 <- svycoxph(Surv(time,status) ~ HcyQ,Total2_nhs) %>% reg_table(round = 3)

p4trend(fit = f4,'HcyQ')

#model2

f5 <- svycoxph(Surv(time,status) ~ Hcy1 +age+sex+reth1+education,Total2_nhs,) %>% reg_table(round = 3)

f6 <- svycoxph(Surv(time,status) ~ HcyQ +age+sex+reth1+education,Total2_nhs,) %>% reg_table(round = 3)

p4trend(fit = f6,'HcyQ')

#model3

f7 <- svycoxph(Surv(time,status) ~ Hcy1+age+sex+reth1+education+

Na+Cotinine+bmi+pir1+Hyperlipidemia+CVD+diabetes,Total2_nhs) %>% reg_table(round = 3)

f8 <- svycoxph(Surv(time,status) ~ Hcy1+age+sex+reth1+education+

Na+Cotinine+bmi+pir1+Hyperlipidemia+CVD+diabetes,Total2_nhs) %>% reg_table(round = 3)

p4trend(fit = f8,'HcyQ')

# quant(Total3$Hcy)

Total3$HcyQ <- quant(Total3$Hcy, n = 4,Q = TRUE,round=3)

Total3$HcyQ.median <- quant.median(Total3$Hcy, n = 4,round=3)

Total3_nhs <- svy_design(Total3)

#moldel1

f9 <- svycoxph(Surv(time,status) ~ Hcy1,Total3_nhs) %>% reg_table(round = 3)

f10 <- svycoxph(Surv(time,status) ~ HcyQ,Total3_nhs) %>% reg_table(round = 3)

p4trend(fit = f10,'HcyQ')

#model2

f11 <- svycoxph(Surv(time,status) ~ Hcy1 +age+sex+reth1+education,Total3_nhs,) %>% reg_table(round = 3)

f12 <- svycoxph(Surv(time,status) ~ HcyQ +age+sex+reth1+education,Total3_nhs,) %>% reg_table(round = 3)

p4trend(fit = f12,'HcyQ')

#model3

f13 <- svycoxph(Surv(time,status) ~ Hcy1+age+sex+reth1+education+

Na+Cotinine+bmi+pir1+Hyperlipidemia+CVD+diabetes,Total3_nhs) %>% reg_table(round = 3)

f14 <- svycoxph(Surv(time,status) ~ HcyQ+age+sex+reth1+education+

Na+Cotinine+bmi+pir1+Hyperlipidemia+CVD+diabetes,Total3_nhs) %>% reg_table(round = 3)

p4trend(fit = f14,'HcyQ')
